# The Effect of Berberine, a Drug From Chinese Folk Medicine, on Serum and Urinary Uric Acid Levels in Rats With Hyperuricemia

**DOI:** 10.7759/cureus.13186

**Published:** 2021-02-07

**Authors:** Hira Naz, Sidra Naz, Rabab Miraj, Akfish Zaheer, Nada Azam, Isham Saleem Mughal, Abdul Wali Khan, Muhammad Ishaq, FNU Sundas, Muhammad Hanif

**Affiliations:** 1 Internal Medicine, Post Graduate Medical Institute, Lahore General Hospital, Lahore, PAK; 2 Internal Medicine, University of Health Sciences (UHS), Lahore, PAK; 3 Internal Medicine, Faisalabad Medical University, Faisalabad, PAK; 4 Internal Medicine, University College of Medicine and Dentistry, Lahore, PAK; 5 Internal Medicine, Services Institute of Medical Sciences, Lahore, PAK; 6 Internal Medicine, College of Physician and Surgeons Pakistan, Peshawar, PAK; 7 Internal Medicine, Hayatabad Medical Complex Peshawar, Peshawar, PAK; 8 Internal Medicine, Khyber Teaching Hospital, Peshawar, PAK; 9 Internal Medicine, Khyber Medical College, Peshawar, PAK; 10 Internal Medicine, Hayatabad Medical Complex, Peshawar, PAK

**Keywords:** hyperuricemia, berberine, allopurinol, potassium oxonate

## Abstract

Background

The principal manifestation of hyperuricemia is gout. Many drugs are in use nowadays to treat gout, but they are linked with multiple side effects. The present study observed berberine (from Chinese folk medicine) on serum and urinary uric acid levels in rats with potassium oxonate-induced hyperuricemia.

Materials and methods

Thirty-six adult healthy female Sprague Dawley rats were randomly divided into six groups of six rats each. To induce hyperuricemia, all the groups except Group A were given potassium oxonate (250 mg/kg) intraperitoneally on days 1, 3, and 7. Group A, the normal control group, was given normal saline for seven consecutive days intraperitoneally. Group C was administered allopurinol (5 mg/kg body weight) intraperitoneally, and Group D, E, and F were given berberine in doses of 0.75 mg/kg, 1.25 mg/kg, and 2.5 mg/kg body weight respectively intraperitoneally for seven consecutive days, one hour after the potassium oxonate injection. On zero, first, third, and seventh day of the experiment, blood and urine samples were taken to estimate the serum and urinary uric acid levels. On days zero and 7, serum uric acid was measured by cardiac puncture, while on days 1 and 3, it was measured by the tail prick method. The uric acid was measured by an enzymatic colorimetric method and creatinine by the Jaffe method. Fractional excretion of urate was also calculated.

Results

Berberine lowered serum uric acid levels in rats with potassium oxonate-induced hyperuricemia with highly significant results (p-value <0.001) in all three dosages. Berberine increased the urinary uric acid level and the fractional excretion of urate in a time-dependent manner in all three dosages. This effect was maximally shown by low dose berberine with a highly significant result (p-value <0.001).

Conclusion

Berberine successfully decreased the serum uric acid level of hyperuricemic rats by increasing the urinary uric acid level and fractional excretion of urate.

## Introduction

Hyperuricemia refers to uric acid levels abnormally high in the blood. Serum uric acid’s normal upper level in men is 7 mg/dl and in women is 6 mg/dl [1]. A raised level of uric acid is the main key factor for gout development and has been linked to renal dysfunction, chronic nephropathy, and nephrolithiasis [2]. Gout is the disorder of inflammatory arthritis, and it manifests as an acute attack initially but can also present as chronic arthritis. Monosodium urate (MSU) crystal formation occurs due to elevated serum uric acid levels above their saturation point [3]. The prevalence of hyperuricemia is the same in both genders, but gout is more common in males [4].

Berberine is an isoquinoline alkaloid present in rhizome, roots, and stem barks of many medicinal plant species distributed widely in nature. In Gilgit-Baltistan, Hindukush, and Karakoram territories of Pakistan, various genus Berberis species (family Berberidaceae) are found [5]. Berberine is primarily investigated and found to possess many biological and pharmacological actions [6].

To lower uric acid levels in gout and hyperuricemia treatment, the first modality employed in the past was increasing uric acid excretion, also known as uricosuric therapy. It was the only pharmacological therapy available until allopurinol (a xanthine oxidase inhibitor) was introduced and became first-line therapy [7]. Uricosuric agents include probenecid, sulfinpyrazone, and benzbromarone. All of these agents’ use cause significant side effects in the body. Therefore, there is room for better pharmacological treatment strategies with lesser side effects, and berberine was investigated for its role in overcoming hyperuricemia.

Chinese polyherbal preparations containing herbs which have berberine as an active principal constituent or berberine itself, have shown an anti-hyperuricemic effect in various animals [8-10]; one human study using berberine in combination with milk thistle, also reduced the serum uric acid level in gouty patients [11, 12].

As a principal constituent, berberine has shown an anti-arthritic effect in vivo [13] and an anti-inflammatory role in monosodium urate crystal-induced inflammation in vitro [14]. A study was conducted at Post Graduate Medical Institute (PGMI) on healthy female Sprague Dawley rats in which berberine was administered daily for a period of 24 weeks to assess the safety of therapeutic dose. At the end of the study, serum uric acid level was significantly lowered in treated rats compared to the non-treated group [15].

By considering all these observations, this study idea was taken to investigate berberine for having a role in overcoming hyperuricemia specifically.

## Materials and methods

Adult healthy female Sprague Dawley rats of bodyweight 140-190 grams were included in the study. Following optimum hygienic conditions, they were caged in Post Graduate Medical Institute’s animal house, Lahore. The animals were kept under natural light and dark cycle and were allowed to acclimatize for one week, according to the standard laboratory conditions (temperature: 25 ± 2°C). Their diet consisted of rat chow and water ad libitum. The experimental protocols, animal care, and handling were according to the criteria mentioned in ‘Guide for the Care and Use of Laboratory Animals.’

The potassium oxonate-induced hyperuricemic animal model was used to study the test agent’s lowering effect on the uric acid level. Potassium oxonate, uricase inhibitor (250 mg/kg) dissolved in 0.9% saline solution, was administered intraperitoneally to each rat, one hour before administering test compounds on first, third, and seventh days of experiment [16].

Six experimental groups were taken, each containing six rats. In group A (Normal control), normal saline was given to all rats once daily by intraperitoneal route for seven consecutive days as a single morning dose in an amount equivalent to that given to the experimental group. In group B (Disease control), rats were given normal saline on days of induction of hyperuricemia, i.e., Day 1, 3, and 7; normal saline was given one hour after potassium oxonate injection. Berberine (purity >99.98%) was purchased from Sigma-Aldrich (St. Louis, MO, USA). Three doses of berberine, 0.75 mg/kg, 1.25 mg/kg, and 2.5 mg/kg, were administered intraperitoneally daily as a single dose from day 1 to day 7 (Group D, E, and F, respectively) [17]. On days of induction of hyperuricemia, i.e., 1, 3, and 7, it was administered one hour after potassium oxonate injection. Allopurinol (pharmaceutical grade) was obtained from the Schazoo Pharmaceutical Laboratories (Pvt.) Ltd (Lahore, Pakistan). A dose of 5 mg/kg was administered intraperitoneally daily as a single dose from day 1 to day 7 to group C (Standard drug). On days of induction of hyperuricemia, i.e., 1, 3, and 7, it was administered one hour after potassium oxonate injection.

Blood sampling

Blood was obtained from the tail vein of rats after three hours of administering test agents on the first and third days of the experiment. At the start of the experiment, i.e., day 0, and at the end of the experiment, i.e., day 7, 1 ml blood was taken through cardiac puncture under light anesthesia. The blood was allowed to clot at room temperature for approximately one hour and then centrifuged at 2500 rpm for 10 minutes. Sera were separated and stored at -20°C to keep it stable for long durations [18]. Results were run by defrosting the samples and warming them up to 37°C. Special attention was given to cloudy urine samples; they were warmed at 37°C for a considerable time to dissolve all the precipitated urate crystals and uric acid [19]. On days 1 and 3, uric acid was measured using a uric acid meter (Multi Sure). On day 0 and day 7, serum uric acid concentrations were measured using a standard diagnostic kit matched to the instrument by the enzymatic colorimetric method [18]. On day 0 and day 7, serum creatinine levels were measured using a standard diagnostic kit matched to the instrument by the Jaffe method.

Urine sampling

On day 0, 1, 3, and 7 of the experiment, three hours of urine were collected after administering the test agent. Urinary uric acid and creatinine levels were measured using a standard diagnostic kit matched to the instrument by enzymatic colorimetric and Jaffe method, respectively. The following formula calculated fractional excretion of urate (FEUa):

FEUa = (Uric Acid(U) × Cr(S) × 100)/(Uric Acid(S) × Cr(U))

where (S) = serum, (U) = urine and Cr = Creatinine

## Results

Mean serum uric acid level ± standard deviation of all the groups is shown in Table 1.

**Table 1:** Effect of different doses of berberine on serum uric acid level (mg/dl) of hyperuricemic rats (n = 6)

Serum Uric Acid			
Groups	Day 0 Mean ± SD	Day 7 Mean ± SD	Paired t-test p-value
Normal control (A)	1.8 ± 0.3	1.9 ± 0.3	0.102
Disease control (B)	1.9 ± 0.4	4.6 ± 0.4	<0.001
Standard drug Allopurinol (C)	1.9 ± 0.3	1.8 ± 0.5	0.747
Low dose berberine (D)	1.6 ± 0.3	2.4 ± 0.6	0.032
Medium dose berberine (E)	1.7 ± 0.3	2.7 ± 0.7	0.037
High dose berberine (F)	1.8 ± 0.2	2.9 ± 0.3	0.001
p-value (ANOVA)	0.431	0.001	

Serum uric acid was measured on day 0 and day 7 by the enzymatic colorimetric method after collecting blood by cardiac puncture. On day 1 and day 3, serum uric acid was measured by a uric acid meter. The values lower than 3 were not measurable by the uric acid meter. They denoted as 'low.' Therefore, serum uric acid values on day 1 and day 3 were not included in stats analysis.

There was no significant difference in serum uric acid levels between all the groups on day 0 by ANOVA. Potassium oxonate elevated the serum uric acid levels, reaching 4.6 ± 0.4 (p-value <0.001 vs. normal control) on the intervention’s 7th day. Following treatment of the hyperuricemic rats by administering berberine in different dosages resulted in a significant reduction of serum uric acid levels in hyperuricemic rats on day 7, as shown by applying paired t-test. Among the various dosages, it is to be noted that low dose berberine maximally reduced the serum uric acid levels on day 7. Medium dose and high dose berberine also lowered serum uric acid levels significantly but not as much as low dose berberine on day 7. There was an insignificant difference in serum uric acid values at the start and end day of intervention in the allopurinol treated group, which showed that allopurinol reduced serum uric acid values to just as normal.

Mean urinary uric acid level ± standard deviation of all the groups is shown in Table 2.

**Table 2:** Effect of different doses of berberine on urinary uric acid level (mg/dl) of hyperuricemic rats (n = 6) on day 0, 1, 3 and 7.

Urinary Uric Acid (mg/dl)							
	Groups	Day 0 Mean ± SD	Day 1 Mean ± SD	Day 3 Mean ± SD	Day 7 Mean ± SD	Repeated measure ANOVA p-value	
	Normal control (A)	7.4 ± 2.3	7.3 ± 2.3	7.0 ± 1.9	7.2 ± 1.9	0.403	
	Disease control (B)	7.6 ± 2.2	10.9 ± 3.2	12.6 ± 3.0	14.6 ± 2.9	0.067	
	Standard drug Allopurinol (C)	9.4 ± 1.2	11.7 ± 3.5	9.6 ± 3.5	7.8 ± 3.3	0.182	
	Low dose berberine (D)	8.6 ± 3.8	6.9 ± 4.5	20.4 ± 12.7	48.5 ± 10.2	0.028	
	Medium dose berberine (E)	10.1 ± 5.5	6.5 ± 4.7	12.3 ± 6.2	24.7 ± 10.9	0.025	
	High dose berberine (F)	7.2 ± 2.9	4.8 ± 1.9	6.8 ± 2.1	17.1 ± 7.3	0.052	
	p-value (ANOVA)	0.575	0.01	0.007	<0.001		

Repeated measure ANOVA was applied to differentiate the effect of berberine on days 0, 1, 3, and 7. Table 2 shows urinary uric acid values remained the same in the normal control group at the start and the end of the study. Potassium oxonate increased the urinary uric acid excretion gradually from day 0, 1, 3 to day 7, but the difference was insignificant. Standard drug allopurinol increased the urinary uric acid excretion from day 0 to day 1. It then decreased from day 1 to day 3 and day 3 to day 7 insignificantly, as shown by repeated measure ANOVA. Berberine-treated groups increased the urinary uric acid excretion in all the dosages with a significant difference from the normal control. First, the urinary uric acid excretion was decreased from day 0 to day 1 and then increased from day 1 to day 3 and exceptionally from day 3 to day 7 in all the berberine-treated groups. Maximum urinary uric acid excretion was observed on day 7 in all the groups, with a highly significant difference shown by ANOVA (p-value <0.001). Among the various berberine-treated groups, low dose berberine showed maximum urinary uric acid excretion on day 7 with a highly significant difference (p-value <0.001). This shows that berberine affects hyperuricemic rats in a dose and time-dependent manner.

Mean feed (fractional excretion of urate) ± standard deviation of all the groups is shown in Table 3.

**Table 3:** Effect of different doses of berberine on fractional excretion of urate (%) of hyperuricemic rats (n = 6).

Fractional Excretion of Urate (FEUa)%			
Groups	Day 0 Mean ± SD	Day 7 Mean ± SD	Paired t-test p-value
Normal control (A)	6.7 ± 4.2	6.8 ± 3.4	0.367
Disease control (B)	6.3 ± 1.7	9.3 ± 4.9	0.188
Standard drug Allopurinol (C)	7.3 ± 1.7	6.4 ± 4.7	0.545
Low dose berberine (D)	9.6 ± 7.7	35.4 ± 13.2	0.007
Medium dose berberine (E)	9.3 ± 3.9	17.4 ± 9.2	0.119
High dose berberine (F)	5.8 ± 3.1	9.4 ± 5.4	0.154
p-value (ANOVA)	0.512	<0.001	

Due to the difference in methods of serum uric acid measurements on different days of study design, only day 0 and day 7 serum uric acid values can be used by applying stats analysis. Hence FEUa was also calculated only on day 0 and day 7 with the following formula's help.

FEUa = (Uric Acid(U) × Cr(S)×100)/(Uric Acid(S) × Cr(U))

Comparing the FEUa values at day 0 and day 7 by different groups follows the same pattern as shown by the urinary uric acid excretion in Table 2. The group having increased urinary uric acid excretion also has a higher fractional excretion of urate. Table 3 shows that FEUa remained the same in normal control, increased disease control, and decreased in the standard allopurinol group with an insignificant difference. After berberine administration, following treatment increased the FEUa in hyperuricemic rats in all three dosages on day 7, but a significant difference was present with the low dose berberine only.

## Discussion

Hyperuricemia, referred to as raised uric acid levels inside the body, has been known for causing many pathological conditions and a significant risk factor for gout [20]. Treatment and prevention of gout and hyperuricemia consist of decreasing uric acid levels inside the body. This process is accomplished by decreasing the production or increasing the excretion of uric acid. Allopurinol and febuxostat, xanthine oxidase inhibitors, and probenecid, a uricosuric agent, are the current treatment modalities for hyperuricemia and gout to reduce the level of uric acid level inside the body [21]. Allopurinol is the clinically preferred drug that is in use nowadays. But it has many side effects such as bone marrow suppression, severe cutaneous reaction, gastrointestinal intolerance, and interstitial nephritis [22]. So there has always been room for more effective treatment modalities.

Chinese polyherbal preparations such as Sanmiao wan, Ermiao wan, and modified Simiao decoction containing herbs which have berberine as an active principal constituent or berberine itself, have shown an anti-hyperuricemic effect in various animals [8-10], as well as one human study using berberine in combination with milk thistle, also reduced the serum uric acid level in gouty patients [11]. As a principal constituent, berberine has shown an anti-arthritic effect in vivo [13] and an anti-inflammatory role in monosodium urate crystal-induced inflammation in vitro [14]. A study was conducted at PGMI on healthy female Sprague Dawley rats in which berberine was administered daily for a period of 24 weeks to assess the safety of therapeutic dose. At the end of the study, serum uric acid level was significantly lowered in treated rats compared to the untreated group [15]. So the results of all these studies support present research, which specifically tested berberine for its hypouricemic action as a sole agent for the first time.

The first objective of the study was to make a model of hyperuricemia. Mostly there are two basic mechanisms employed to induce hyperuricemia in rats. One method utilizes yeast extract powder or other purine diets, which cause increased production of uric acid inside the rat. The other method utilizes potassium oxonate, which causes decreased decomposition of uric acid by inhibiting enzyme urate oxidase or uricase (lacks in humans) competitively and selectively [23]. This animal hyperuricemic model artificially and temporarily elevates uric acid levels by blocking uric acid conversion to allantoin [24].

In this study, potassium oxonate was given in a dose of 250 mg/kg intraperitoneally on days 1, 3, and 7 to make a hyperuricemia model to all groups except group A (the normal control group). This model was successful in raising the serum uric acid level with a highly significant result as compared to normal control (p-value <0.001) and also slightly raised urinary uric acid level with an increase in fractional excretion of urate that is due to the increase of plasma uric acid level and a greater amount of uric acid filtered in the kidney tubules [25]. These results are comparable to Shah and Shah’s study in which a similar model showed the same effect on serum, urinary uric acid level, and its fractional excretion [26].

The test compound berberine at different dosages of 0.75 mg/kg, 1.25 mg/kg, and 2.5 mg/kg was given intraperitoneally for seven consecutive days to hyperuricemic rats. It statistically decreased the serum uric acid level on day 7 with highly significant results (p-value <0.001 vs. disease control) and also increased the urinary uric acid level and fractional excretion of urate in all dosages. Allopurinol, a standard drug, was given to group C; it statistically decreased the serum uric acid level and the urinary uric acid excretion and fractional excretion of urate. By comparing the effect of berberine to allopurinol (standard drug) in overcoming hyperuricemia, it was found that serum uric acid level was lower with allopurinol than berberine. Still, the difference was significant only with a high dose. Low and medium doses had insignificant higher levels. As allopurinol is a principal xanthine oxidase inhibitor, it reduced the production of serum uric acid. As enough uric acid was not present inside the body, the urinary uric acid level also decreased along with decreased fractional excretion of urate, as shown in another study also [23]. In contrast to allopurinol, berberine, the test drug, increased the urinary uric acid level and fractional excretion of urate, which suggested that it has more roles on renal transporters and uricosuric action than xanthine oxidase inhibition. On comparison of serum uric acid level between day 0 and 7, it is observed that allopurinol reduced the uric acid level to baseline, which berberine failed to do so. It is also observed that numerically uric acid level is lowest with the low dose among the berberine-treated groups. If a dose lower than 0.75 mg/kg had been used, it might have produced a better result.

Berberine reduced the serum uric acid level and increased the urinary uric acid level and fractional excretion of urate. Berberine followed a time-dependent fashion of urinary uric acid excretion. The urinary uric acid level decreased on day 1 and then increased gradually on day 3 and reached maximum levels on day 7. All doses followed this pattern. The low dose berberine (0.75 mg/kg) showed the maximum urinary excretion in a time-dependent manner with highly significant results of p-value <0.001 and consequently maximum fractional excretion of urate (p-value <0.001) as compared to disease control. The high dose showed the minimum urinary uric acid excretion and fractional excretion of urate among the three doses.

To comment on the possible mechanism involved behind this uricosuric berberine action could be that urate transporters are basically involved in uric acid reabsorption and secretion in the kidney tubules. URAT1 and GLUT9 are the major transporters that mediate uric acid reabsorption in the proximal tubules and are known as the main therapeutic targets for considering hyperuricemia and gout. Most of the available uricosuric drugs such as benzbromarone, sulfinpyrazone, and probenecid act by inhibiting these transporters [27]. So further research is needed to know how berberine affects these kidney transporters.

The present study results show that berberine successfully reduces the serum uric acid level in hyperuricemic rats. Berberine possesses a specific property of antioxidant action, as confirmed by various studies. The higher level of uric acid in humans by losing the uricase gene in an evolutionary process has been thought to be a defensive mechanism for a longer life span [28]. The reason for this behavior is that uric acid itself possesses antioxidant properties. Therapeutic agents that decrease uric acid levels in hyperuricemic states are also devoid of uric acid's useful property. The use of this herbal compound berberine as a hypouricemic agent will have added benefit as it can decrease the serum uric acid level. It can also compensate for the attenuated antioxidant action related to low uric acid levels inside the body.

## Conclusions

Based on the present study results, it is concluded that berberine lowers serum uric acid levels by increasing urinary uric acid excretion in rats with potassium oxonate-induced hyperuricemia. All three doses showed this effect; low, medium, and high dose berberine. Among the three doses, low dose berberine showed the maximum anti-hyperuricemic action by maximally increasing the urinary uric acid excretion and fractional excretion of urate.
